# Cacao sustainability: The case of cacao swollen-shoot virus co-infection

**DOI:** 10.1371/journal.pone.0294579

**Published:** 2024-03-07

**Authors:** Folashade B. Agusto, Maria C. A. Leite, Frank Owusu-Ansah, Owusu Domfeh, Natali Hritonenko, Benito Chen-Charpentier

**Affiliations:** 1 Department of Ecology and Evolutionary Biology, University of Kansas, Lawrence, Kansas, United States of America; 2 Department of Mathematics and Statistics, University of South Florida St Petersburg, St Petersburg, Florida, United States of America; 3 Social Science and Statistics Unit, Cocoa Research Institute of Ghana, New Tafo-Akim, Eastern Region, Ghana; 4 Pathology Division, Cocoa Research Institute of Ghana, New Tafo-Akim, Eastern Region, Ghana; 5 Department of Mathematics, Prairie View A&M University, Prairie View, Texas, United States of America; 6 Department of Mathematics, The University of Texas at Arlington, Arlington, Texas, United States of America; Kwame Nkrumah University of Science and Technology, GHANA

## Abstract

The cacao swollen shoot virus disease (CSSVD) is among the most economically damaging diseases of cacao trees and accounts for almost 15–50% of harvest losses in Ghana. This virus is transmitted by several species of mealybugs (Pseudococcidae, Homoptera) when they feed on cacao plants. One of the mitigation strategies for CSSVD investigated at the Cocoa Research Institute of Ghana (CRIG) is the use of mild-strain cross-protection of cacao trees against the effects of severe strains. In this study, simple deterministic, delay, and stochastic ordinary differential equation-based models to describe the dynamic of the disease and spread of the virus are suggested. Model parameters are estimated using detailed empirical data from CRIG. The modeling outcomes demonstrate a remarkable resemblance between real and simulated dynamics. We have found that models with delay approximate the data better and this agrees with the knowledge that CSSVD epidemics develop slowly. Also, since there are large variations in the data, stochastic models lead to better results. We show that these models can be used to gain useful informative insights about the nature of disease spread.

## 1. Introduction

Vector-borne plant viruses cause a wide range of diseases in plants with grave economic consequences [1]. A recent increase in the spread of plant pests and diseases is caused by globalization, climate change, agricultural intensification, and reduced resilience in production systems [2]. A vast number of plant pathogens pose a serious threat to food safety and security, national economies, biodiversity, and rural environment. An example of such pathogens is the cacao swollen shoot virus (CSSV), the causal agent of the cacao swollen shoot virus disease (CSSVD) [3, 4].

CSSVD was first observed in the Eastern Region of Ghana in 1936 by a farmer and its virus nature was confirmed in 1939 [5]. CSSVD is considered the most economically damaging cacao virus disease that could account for 15–50% yield loss if the severe strains are involved in infections. CSSV is classified as a member of the plant-infecting pararetroviruses in the genus *badnaviridae* which are with non-enveloped bacilliform particles that encapsulate a circular double-stranded DNA-genome. Previously, the isolates and strains were grouped according to the severity of symptom expression and geographical origin. Now, it is known that CSSVD is caused by a complex of badnavirus species based on their molecular structure [6].

The virus affects all parts of the cacao plant [5]. The symptoms seen on the leaves include red vein banding of the immature “flush” leaves [5, 7], chlorotic vein flecking or banding which may occur in angular flecks, chlorotic vein clearing, and various forms of mosaic symptoms [5]. The virus causes swellings of the stems (nodes, internodes, tips) and roots [5, 7, 8]. Some strains also cause infected pods to change shape and become rounder, smaller and with smoother surfaces.

The cacao swollen shoot virus is semi-persistently transmitted by several species of mealybugs (*Pseudococcidae, Homoptera*) on cacao [9]. The infection occurs when mealybugs acquire the virus from infected cacao or alternative host plants and deposit them in healthy cacao plants during feeding. The mealybug species differ in their ability to transmit different strains of the virus. The most efficient mealybug CSSV transmitters including the *Formicococcus njalensis* (Laing), *Planococcus citri* (Risso), and *Ferrisia virgata* (Okll) are also dominant on cacao fields in Ghana and Cote d’Ivoire [10].

In the past, several mitigation measures were proposed to curtail the spread of the virus, such as cutting-out of infected trees [9, 10] and breeding for resistant trees [9]. Between 1946 and 1948, over 254 million cacao trees were lost in Ghana as a result of the cutting-out campaign schemes initiated by the Cocoa Health and Extension Division of Ghana Cocoa Board [9, 10]. The cutting-out campaign has faced several challenges including farmer resistance, land tenure issues, and discontinuity in official policy on CSSVD management. Mild strain cross-protection has been considered as one of the alternative management options [10–12].

Our aim in this study is to use simple differential equation models to gain useful insights about the inherent dynamics of an experimental data involving cacao swollen shoot virus from Domfeh *et al.* [10]. Several studies have used mathematical models to study diseases transmission dynamics in plant [12–18], including co-infection with multiple pathogen and the resulting interactions like cross protection [12] and helper-dependent [18]. Mathematical models have also been used to understand mitigation strategies to curtail the spread of some plant pathogen [17, 19]. Thus, we develop several models using differential equations to determine the disease transmission rate in experimental treatments with and without protective layers. We also develop models with stochasticity to capture the noise in the data. To the best of our knowledge, these models are the first mathematical models specifically developed for CSSV; they are also the first models to mathematically assess the effect of the crop protective layers using delay and stochastic differential equations.

## 2. Methodology

### 2.1 Experimental data

Data on CSSVD infection under varied protection conditions was required for the study. An available data set from an experimental study in Ghana was used [10]. The experiment involved 4 treatments laid out in a randomized complete block design with three replications. The plots were designed based on the nature of the treatments. The plots used were squares made up of 19 by 19 trees planted at a spacing of 2.4 meter square. This resulted in a 10 nested squares made up of perimeter trees ranging from one tree for the 10^*th*^ square (i.e., at the centre) to 72 trees for the first square (outer perimeter). All the plots had the first two perimeter trees inoculated with CSSV-1A severe strain to serve as a source of inoculum. The main attribute that differentiated the treatments were the number of perimeter trees along the 3^*rd*^ to the 10^*th*^ nested squares, which were protected against the CSSV-1A using the mild strain N1. The first treatment (*T*_1_) had the 3^*rd*^ to the 7^*th*^ perimeters inoculated with N1 strain. The second treatment (*T*_2_) had the 3rd perimeter to the 5^*th*^ perimeter plants inoculated with N1. The third treatment (*T*_3_) had none of the trees inoculated with the mild strain, while the fourth treatment (*T*_4_) had all the 3rd to 10^*th*^ perimeter trees inoculated with mild N1 strain. Mild strain inoculation was done prior to transplanting of the cacao seedlings. After transplanting, the spread of the CSSV-1A among the 3^*rd*^ to 10^*th*^ perimeter trees was monitored for 7 years. The data obtained in Domfeh *et al.* [10] are repeated below.

#### Treatment data *T*_1_, *T*_2_, *T*_3_

The experimental data obtained from Domfeh *et al.* [10] for tree level spread of CSSV for treatments *T*_1_, *T*_2_, and *T*_3_ are given in Tables 1–3 below.

**Table 1:** Treatment *T*_1_.

*T*_1_	Number of trees with N1 in rows 1–5 infected with 1A	Number of trees with N1 in row 1–5	Number of susceptible trees in rows 6–8 infected with 1A	Number of susceptible trees in rows 6–8
Year 1	0	600	0	75
Year 2	1	599	0	75
Year 3	130	470	6	69
Year 4	137	463	6	69
Year 5	151	449	7	68
Year 6	177	423	22	53
Year 7	202	398	28	47

**Table 2:** Treatment *T*_2_.

*T*_2_	Number of trees with N1 in rows 1–3 infected with 1A	Number of trees with N1 in row 1–3	Number of susceptible trees in rows 4–8 infected with 1A	Number of susceptible trees in rows 4–8
Year 1	0	423	0	240
Year 2	7	425	0	240
Year 3	129	306	27	213
Year 4	131	301	30	210
Year 5	136	296	33	207
Year 6	169	263	66	174
Year 7	193	239	78	162

**Table 3:** Treatment *T*_3_.

*T*_3_	Number of trees with N1 in rows 1–8 infected with 1A	Number of Susceptible trees
Year 1	2	670
Year 2	14	658
Year 3	151	521
Year 4	198	474
Year 5	208	464
Year 6	260	412
Year 7	310	362

### 2.2 Model formulation

In this section, we develop mathematical models for a description of the transmission of CSSV infection in cacao trees. We propose four models; two are deterministic models and the other two are stochastic models. With these two types of models, one incorporates delay while the other does not. Briefly, deterministic models assume that known average rates with no random deviations are applied to large populations and delay models assume that the derivative of the unknown function at a given time is defined in terms of the values of the function at previous times [20, 21].

Before describing the models, we state the following assumptions used in their formulation.

#### Assumptions

(i) Based on knowledge in the field, mealybugs transmit the virus neither to other mealybugs nor to their off-spring [22, 23]. The mode of transmission of the virus to cacao trees is in a semi-persistent manner [22].
(ii) Infection in the mealybugs does not cause additional mortality of the vectors, and the viral load in the mealybugs will be cleared approximately 72 hours after CSSV is acquired [24].
(iii) The agents responsible for infecting trees are viruliferous mealybugs. The viral load of cacao trees infected with mild strains is lower compared to infection with severe strains. The probability of susceptible trees being naturally infected with mild strain is therefore very low [11]. Therefore, in our model, we consider that the infection of susceptible trees with mild strain (N1) in the field is negligible.
(iv) We denote the average number of mealybugs carrying severe strain per infected tree as *J*. Based on expert knowledge in the field, we use *J* = 20 in our numerical simulations [25]. Our results are still applicable to a wide range of *J* obtained from experimental data. In reality, there is some randomness associated with this quantity and our stochastic models include this assumption in an implicit form. The randomness inherent in the number of viruliferous mealybugs per tree could be included explicitly by assuming that it follows, for example, a normal distribution.
(v) If a mealybug is found on a severely infected cacao tree, it is assumed to be infected with the severe strain 1*A*. That is, the infection is instantaneous. This is reasonable because the time scale of our model is one year and the time scale of infection by mealybugs is in hours.
(vi) The experimental data in [10] suggests that there is some delay in the spread of the infection into a tree after being exposed to virus-carrying mealybugs, and also it takes some time before the symptoms can be detected. Based on the data we assume that the delay is 1 year.
(vii) Mealybugs have several ways of movement, like moving from canopy-to-canopy or being carried by attendant ants or by the wind. However, the rates of movement of the first two (canopy-to-canopy and ants) are so slow (particularly, when the canopy is not closed), that the mealybugs can lose their infectivity before getting to a healthy tree. Therefore, in this work, we assume that the only way for infected mealybugs to cross a barrier of mild strain *N*1-inoculated trees is when the mealybugs are carried by the wind. Hence, for simplicity, we will not consider the spatial distribution of the trees but we will implicitly incorporate space via a parameter representing the disease transmission probability.
(viii) The effect of the wind carrying viruliferous mealybugs across inoculated barriers is random. This randomness can be included in different ways but since the probability distributions of the strength, the direction of the wind, etc. are unknown, a common method is to model such randomness by introducing white noise into the model.

To formulate the model, we segment plant population according to their disease status. The number of mildly infected trees (infected with strain *N*1) is denoted by *Y* and the number of healthy cacao plants is denoted by *X*, the number of severely infected (infected with strain 1*A*) cacao trees is represented by *Z*. Since the total number of trees and the number of mildly infected trees per plot are constant, it is sufficient to consider only one population, either susceptible or severely infected trees. We will use susceptible trees.

Based on assumptions (i)-(vii) we describe the vector population disease dynamics by the evolution of the number of mealybugs infected with the strain 1*A*, which is assumed to be proportional to the number of severely infected trees. Following (iv) we take this to be *JZ*.

Based on the above considerations and hypothesis, the resulting deterministic and stochastic models describing the CSSV disease transmission in healthy cacao trees are given in the Eqs (1)–(9) below.

#### 2.2.1 Deterministic model

In this section, we present two types of deterministic models, the model without and with delay.

*Deterministic model without delay*. As mentioned in the previous section, the CSSV transmission can be described by an ordinary differential equation modelling the dynamics of susceptible trees *X*:

$$\begin{array}{c} \frac{dX}{dt}=-pJZ\left(t\right)X\left(t\right), \end{array}$$ (1)

where *J* is the average number of mealybugs per severely infected tree and the parameter *p* represents the transmission probability of 1*A* strain to the susceptible cacao trees. *Z* is the number of severely infected trees in a given plot calculated using the following relation:

$$\begin{array}{cc} Z= & \;\text{total}\;\text{number}\;\text{of}\;\text{initial}\;\text{trees}-\text{number}\;\text{of}\;\text{susceptible}\;\text{trees}-\\ & \;\text{number}\;\text{of}\;\text{mildly}\;\text{infected}\;\text{trees}. \end{array}$$ (2)

Let *N* be the total number of trees, which is constant and note that *Y*, the number of mildly infected trees, is assumed to be constant within each treatment. Thus, the relation (2) can be written as:

$$\begin{array}{c} Z=N-X-Y. \end{array}$$

Linear ODE (1) with the initial condition *X*(*t*_0_) has the solution

$$X\left(t\right)=X\left(t_{0}\right)\text{exp}(-pJ\int_{t_{0}}^{t}Z\left(t\right)dt),$$

or in the explicit form as

$$\begin{array}{c} X\left(t\right)=\frac{\left(N-Y)\text{exp}(-pJ(N-Y)t\right)}{\text{exp}\left(-pJ(N-Y)t\right)-C_{1}}, \end{array}$$ (3)

where *C*_1_ is the constant of integration.

*Deterministic model with delay*. If we consider that the number of infected mealybugs is a deterministic variable and include the assumption that after being bitten by a severely infected mealybug a tree takes some time *τ* to develop the disease and show symptoms (assumption (vi)), then the dynamics of the virus transmission is described by the following delay differential equation:

$$\begin{array}{c} \frac{dX}{dt}=-20pJZ\left(t-\tau \right)X\left(t-\tau \right), \end{array}$$ (4)

where *τ* denotes the delay. The data is reported once a year and suggests that there is a 1 year delay on the on-set of observable infection symptoms. Thus, we consider the delay *τ* to be 1 year (see assumption (vi)) and the units of time *t* and *τ* be taken in years.

The simulation results for models (1) and (4) are given in Section 4.

#### 2.2.2 Stochastic model

In this section, we extend models (1) and (4) to incorporate noise according to assumptions (iv), (viii). The proposed models consist of a stochastic differential equation with white noise to capture the variability observed in the data. The extension of model (1) does not include delay given in Eqs (5) and (6) while the extension of Eq (4) is a delayed stochastic differential equation shown in Eqs (8) and (9). The difference between models (5), (6), (8), and (9) is the type of noise that has been implemented. In the models (5) and (8) an environmental noise is considered while in models (6) and (9) a noise proportional to the number of infected trees is integrated. These models show how the two types of noise can be incorporated separately. We also develop a model where the two random effects are integrated in a combined fashion, which is shown in Eqs (7) and (10).

*Stochastic model without delay*

(i) Stochastic model with environmental noise
  $$\begin{array}{c} dX(t)=-pJZ(t)X(t)dt+\sigma dW, \end{array}$$ (5)
  where *σ* measures the intensity of the additive environmental white noise *dW*.
(ii) Stochastic model with multiplicative white noise *dW* proportional to noise in the number of infected trees
  $$\begin{array}{c} dX(t)=-pJZ(t)X(t)dt+\sigma Z(t)dW. \end{array}$$ (6)
(iii) We can also formulate a model that combines both types of noise. However, we leave this out of our simulation results and discussions. This model is given as
  $$\begin{array}{c} dX\left(t\right)=-pJZ\left(t\right)X\left(t\right)dt+\sigma_{1}dW_{1}+\sigma_{2}Z\left(t\right)dW_{2}, \end{array}$$ (7)
  where *σ*_1_ measures the intensity of the additive environmental white noise *dW*_1_, *σ*_2_ measures the intensity of the multiplicative white noise *dW*_2_ proportional to the number of infected trees.

*Stochastic model with delay.* If we consider that the number of infected mealybugs is a deterministic variable, include the assumption (vi) that there is a delay, and that there is stochasticity associated to the movement of vectors (assumption (vii) and (viii)), then the dynamics of the virus transmission is described by the following delay stochastic differential equations:

(i) Model with delay and additive environmental noise *dW*
  $$\begin{array}{c} dX(t)=-pJZ(t-\tau )X(t-\tau )dt+\sigma dW, \end{array}$$ (8)
  where *σ* denotes the intensity of the white noise *dW*.
(ii) Model with delay and noise in the number of infected trees *Z*
  $$\begin{array}{c} dX(t)=-pJZ(t-\tau )X(t-\tau )dt+\sigma Z(t-\tau )dW. \end{array}$$ (9)
(iii) Model with delay and both types of noise considered in (a) and (b)
  $$\begin{array}{c} dX\left(t\right)=-pJZ\left(t-\tau \right)X\left(t-\tau \right)dt+\sigma_{1}dW_{1}+\sigma_{2}Z\left(t-\tau \right)dW_{2}, \end{array}$$ (10)

Our numerical investigation shows that there is no clear difference in the performances of the two stochastic models (8) and (9) and the corresponding models without delay. Hence, we present here only the models with delay and state the results of the stochastic models without delay. Additionally, the model equations (7) and (10) are presented as an illustrative case of how the environmental noise and the noise proportional to the number of infected trees can be integrated simultaneously. The simulation results for models (8) and (9) are given in S1 and S2 Figs.

## 3. Parameter estimation

We estimate the parameter *p* using treatment *T*_1_, *T*_2_, and *T*_3_ data obtained in Domfeh *et al.* [10] and summarized in Section 2.1 above. For each treatment *T*_1_, *T*_2_, and *T*_3_, the parameter *p* is estimated by fitting model predictions to the treatment data using the least squares regression method [26, 27]. For a fixed treatment, the least squares regression method measures the distance between model predictions and data points at the same time period. It is defined as the sum of point-by-point distances squared between the model prediction and the data given as:

$$\begin{array}{c} S=\sum_{i=1}^{n}{\left(y_{i}-d_{i}\right)}^{2}, \end{array}$$ (11)

where *n* is the number of time points, *y*_*i*_ is the model prediction at time point *i*, and *d*_*i*_ is the data for that time point. The best fitting across many runs with different parameter sets is the one that minimizes the least squares statistic in Eq (11).

The fit is performed with the deterministic models, without and with delay. The parameters are given in Table 4.

**Table 4:** Estimates of parameter *p* obtained from fitting models (1) and (4) to the data from the three experimental treatments *T*_1_, *T*_2_, *T*_3_.

Deterministic Model	Treatment	Parameter *p*
model (1) without delay	*T*_1_	8.8 × 10^−6^
	*T*_2_	5.0 × 10^−6^
	*T*_3_	1.2 × 10^−5^
model (4) with delay	*T*_1_	8.4 × 10^−6^
	*T*_2_	4.8 × 10^−6^
	*T*_3_	1.2 × 10^−5^

## 4. Results

Since there were no observable changes in the number of infected trees in the first year when considering treatment *T*_1_, *T*_2_ and just a slight change when considering treatment *T*_3_, we started our simulations at year 1 but the graphs only depict simulations starting at year 2. For each of the treatment *T*_1_, *T*_2_, *T*_3_, the initial condition for the state variable *X*, was taken to be the corresponding observable value at year 1. That is, the initial conditions used were 75 for *T*_1_, 240 for *T*_2_ and 670 for *T*_3_. All suggested models were solved numerically using a time step of 1 year to be in agreement with the experimental collection of data. A Runge-Kutta-Felhberg method of order 4–5 was implemented in the Matlab routine ode45 [28]. For deterministic delay differential equations, any method used for ordinary differential equations can be adapted by solving the equation in steps of length *τ* where *τ* is the delay [29]. We used the routine DDE23 in Matlab [30] to solve (4). A common method for solving stochastic equations is Milstein’s method [31, 32]. We implemented our own version of this method to solve (5) and (6). For solving the stochastic models with delay (8) and (9), a routine was written combining the method of steps for delay equations with Milstein’s method.

The reported field data has an estimated error of ±5%. The values of *σ*, *σ*_1_, *σ*_2_ in the models were chosen so the majority of the experimental points fall within the 95% confidence interval computed assuming the magnitude of error in the data points is ±5%.

### 4.1 Deterministic model

**Fig 1:**
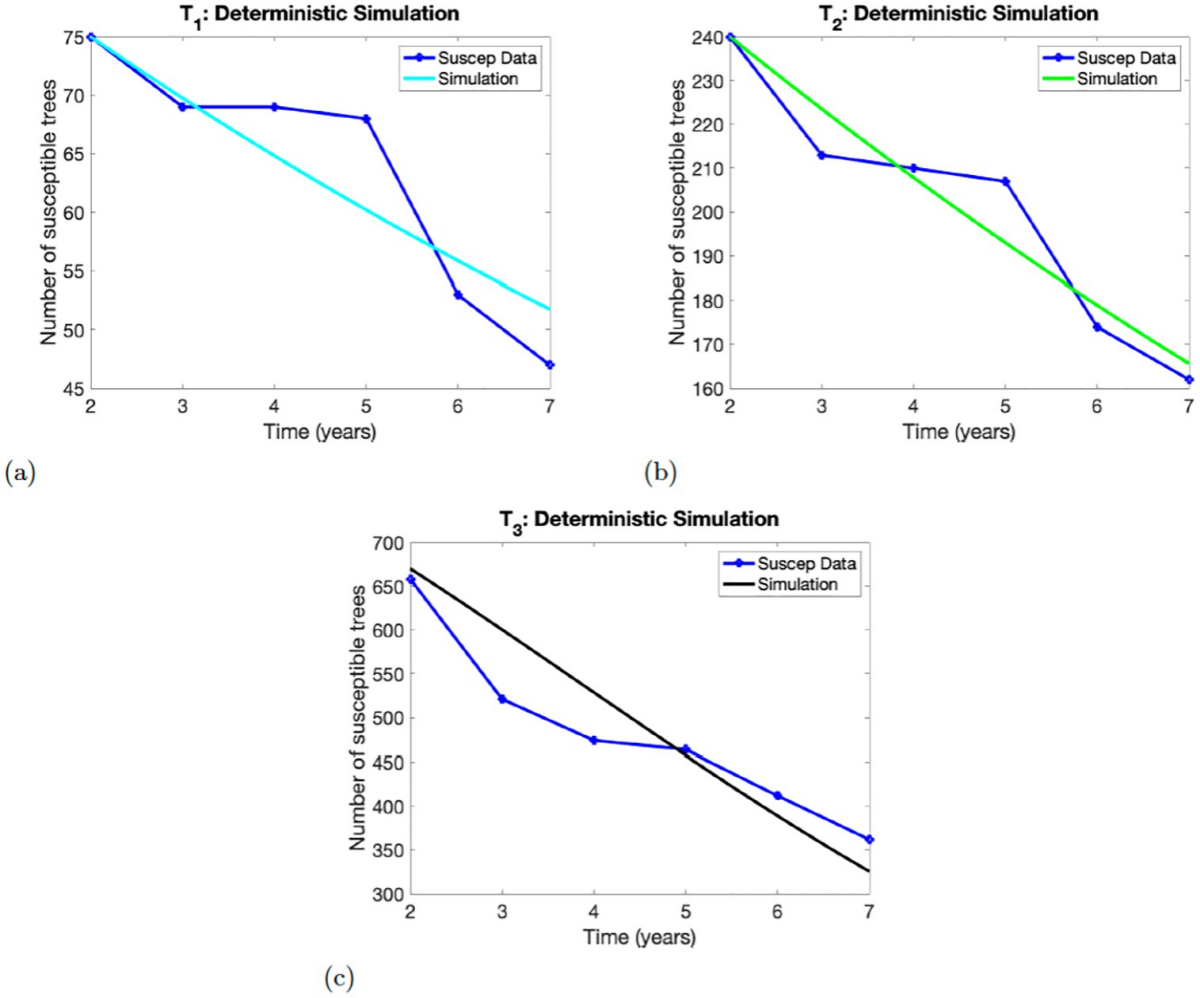
Numerical simulations of the model (1). Panels (a)-(c), show the results for experimental treatment *T*_1_, *T*_2_, *T*_3_, respectively. The initial conditions at *t* = 1 years are *X*(1) = 75 for *T*_1_, *X*(1) = 240 for *T*_2_ and *X*(1) = 670 for *T*_3_.

**Fig 2:**
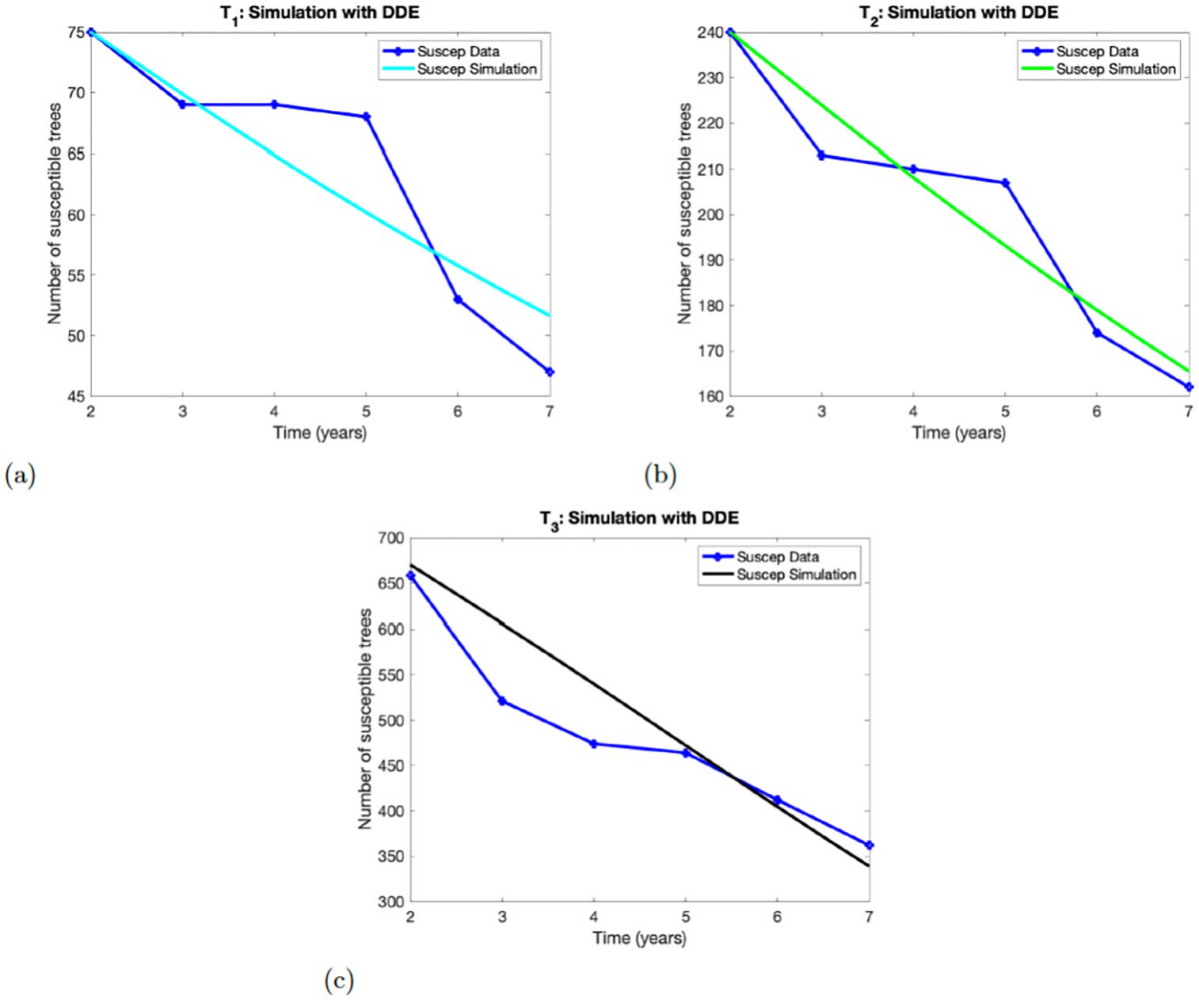
Numerical simulations of the model (4). Panels (a)-(c), show the results for Experimental treatment *T*_1_, *T*_2_, *T*_3_, respectively. The number of susceptible trees at initial time *t* = 1 are *X*(1) = 75 for *T*_1_, *X*(1) = 240 for *T*_2_ and *X*(1) = 670 for *T*_3_.

The simulations of the deterministic models without and with delay, namely models (1) and (2), are depicted in Figs 1 and 2, respectively. These simulations used the estimated values of the parameter *p* given in the Table 4, which are obtained by fitting the models to the data from treatment *T*_1_, *T*_2_, and *T*_3_. The graphs also show the experimental data. In reality the data are discrete points but we use a continuous plot to facilitate the visual comparison with the results obtained from the simulation of the models.

#### 4.1.1 Deterministic model without delay

Fig 1 shows the simulation result using model (1) and the observed data for the three experimental treatments *T*_1_, *T*_2_, *T*_3_.

For treatment *T*_1_, the simulation captured the data in year 2 and year 3. While the simulation for *T*_2_ captured years 2 and 4. Lastly, for treatment *T*_3_, the simulation captured the data points at years 2 and 5. All the simulations captured the data point in year 2 for the three experimental treatments *T*_1_, *T*_2_, *T*_3_.

**Remark 1** *The simulation plots in* Fig 1 *look linear because the infection rate p is very small* (*see* Table 4), *hence simplification of* Eq (3) *using Taylor series expansion is approximately a linear function of the healthy susceptible plant X*. *We also observe this for the simulation figures in the sections below*.

#### 4.1.2 Deterministic model with delay

The simulations of the model (2) using the observed data for the three experimental treatments *T*_1_, *T*_2_, *T*_3_ are depicted in Fig 2. We observe that the simulation results captured the data points in year 2 in both treatments *T*_1_ and *T*_2_ but was close to the data point in year 3 in treatment *T*_1_, while it was close to the year 4 in treatment *T*_2_. In contrast, the simulation results were close to the data points in years 2, 5 and 6 for treatment *T*_3_.

Thus, comparing the solution profile in Figs 1 and 2 shows that model (2) performs better in capturing the data points of the three experimental treatments *T*_3_.

### 4.2 Stochastic model with delay

**Fig 3:**
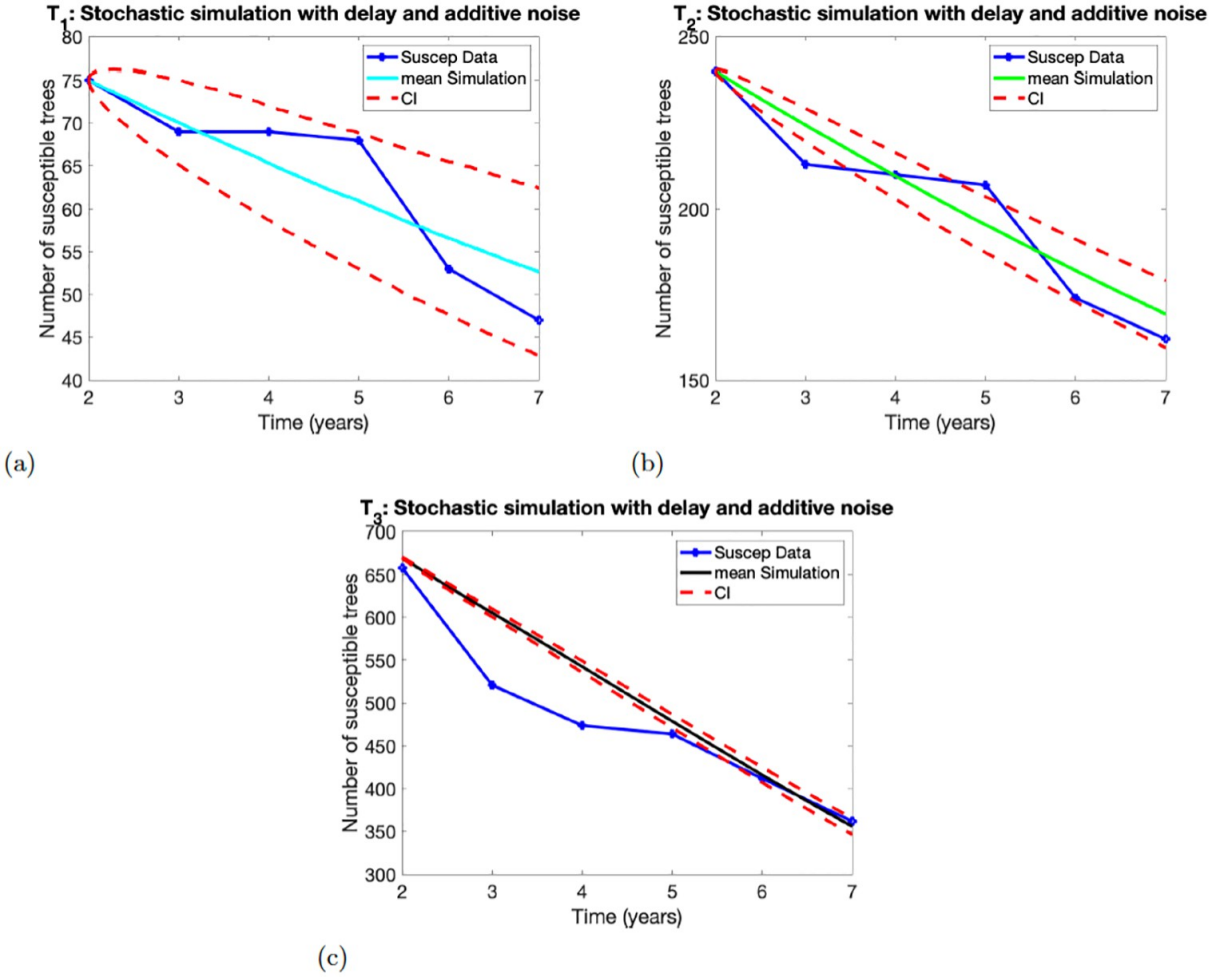
Numerical simulations of the model (8). Panels (a)-(c), show the results for Experimental treatment *T*_1_, *T*_2_, *T*_3_, respectively. The number of susceptible trees at initial time *t* = 1 are *X*(1) = 75 for *T*_1_, *X*(1) = 240 for *T*_2_ and *X*(1) = 670 for *T*_3_.

**Fig 4:**
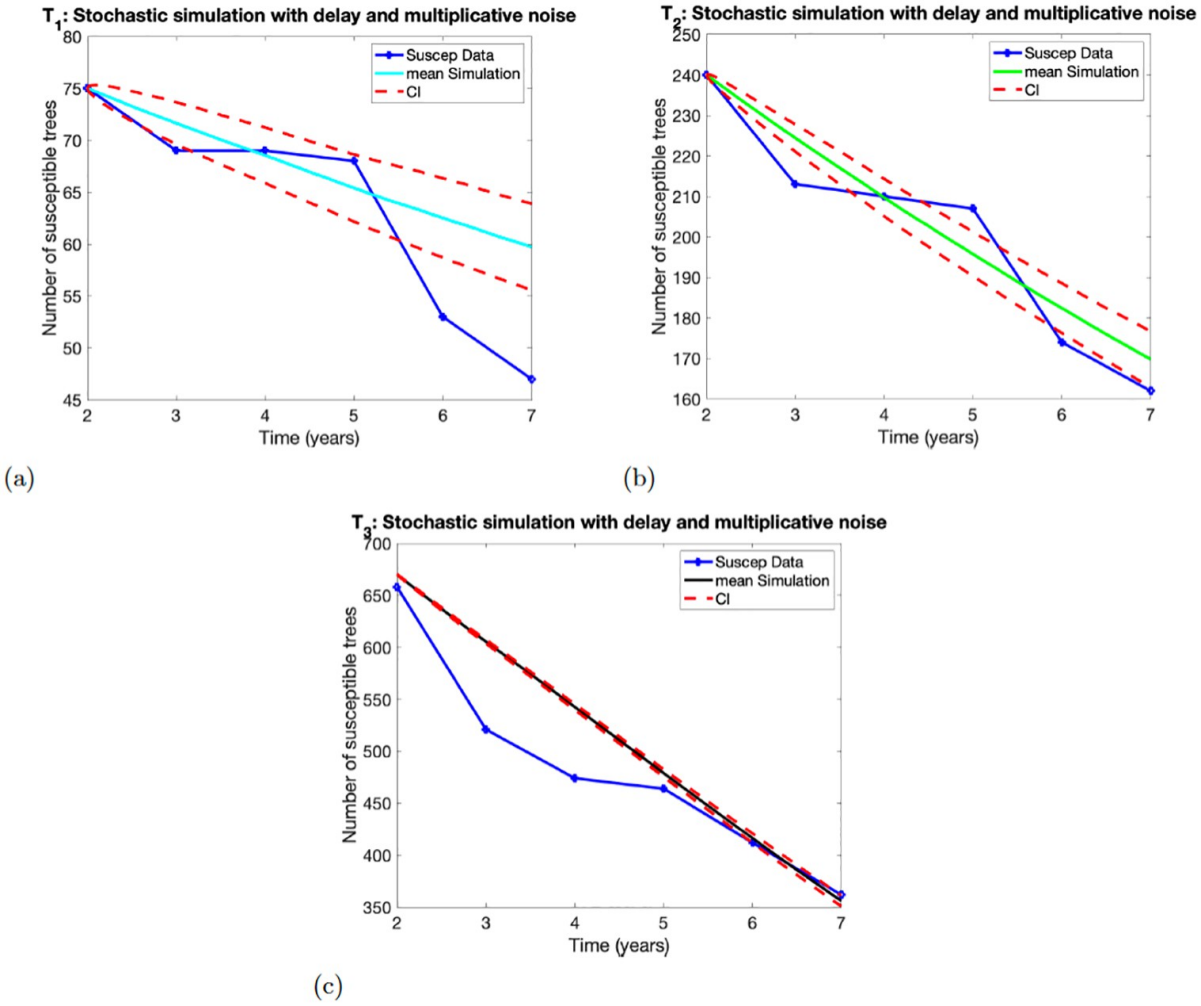
Numerical simulations of the model (9). Panels (a)-(c), show the results for experimental treatment *T*_1_, *T*_2_, *T*_3_, respectively. At initial time *t* = 1, the values of susceptible trees were taken to be *X*(1) = 75 for *T*_1_, *X*(1) = 240 for *T*_2_, and *X*(1) = 670 for *T*_3_.

The simulations of the Stochastic models (8) and (9) with delay coupled with additive and multiplicative noises are depicted in Figs 3 and 4, respectively. The simulations used the estimated parameter *p* given in Table 4.

#### 4.2.1 Stochastic model with delay and additive noise

Fig 3 depicts the simulation results of model (8) using parameter *p* estimated from the three experimental treatments *T*_1_, *T*_2_, *T*_3_ data. For treatment *T*_1_ in Fig 3(a), the simulation captures the data in years 2 and came close to the data point in year 3. However, the confidence interval encompasses all the data points of the treatment. The simulation for *T*_2_ captured years 2 and 4 in Fig 3(b). The confidence interval contains mostly the data points except the data in years 3 and 5. Although these data points are not within the confidence interval, the data points are close to the confidence interval. Lastly, for treatment *T*_3_ in Fig 3(c), the simulation captures the data points at years 6 and 7 and approximates well the data points at years 2 and 5. The confidence interval is thin and only encloses the data points in years 6 and 7.

#### 4.2.2 Stochastic model with delay and multiplicative noise

The simulations of the model (9) using parameter *p* estimated from the three experimental treatments *T*_1_, *T*_2_, *T*_3_ data are depicted in Fig 4. The numeric results of model (9) for treatment *T*_1_ in Fig 4(a), captures the data in year in 2 and is close to the data point in year 4. The confidence interval only contains the data points for years 4 and 5, and the data point in year 3 is close to the confidence interval. In Fig 4(b), The simulation for *T*_2_ captures the data in years 2 and 4. The confidence interval on the other hand contains the data points in years 4 and 7, while the data in years 3, 5 and 6 are close to the confidence interval but not within it. Lastly, for treatment *T*_3_ in Fig 4(c), the simulation captures the data points at years 2, 6 and 7. The confidence interval is also thin and only encloses the data points in years 6 and 7.

The solution profile in Fig 4 looks similar to the solution profile in Fig 3. However, they exhibit some differences. The width of the confidence interval differentiates the solution profiles. The confidence interval in Fig 3 contains all the data points for treatment *T*_1_, but the confidence interval in Fig 4 contains only two data points. We observe similar dynamics for treatments *T*_2_ and *T*_3_. The width of the confidence interval in Fig 4 is smaller than the width of the confidence interval in Fig 3.

## 5. Discussion and conclusions

### 5.1 Discussion

In this study we use two deterministic models without and delay (models (1) and (4)) and two stochastic models ((8), and (9)) with additive and multiplicative noises to capture the infection transmission dynamics of CSSV in cacao tree using three treatment data *T*_1_, *T*_2_, *T*_3_ over a period of 7 years. The data are used to estimate the disease transmission parameter *p* by fitting the models (1) and (4) to data using least squares regression method that minimizes the sum of the squares of point-by-point distances between the model prediction and the data. The output was used in the simulation results in Figs 1–4.

The estimated parameter *p* in treatment *T*_1_ and *T*_2_ have the same order of magnitude. In principle the thicker the barrier the smaller the transmission but that is not the case here. This may be due to the fact that we did not explicitly model the movement of the mealybugs. In our future work we will consider this. In contrast, the estimated parameter *p* in treatment *T*_3_ is higher. This result shows that the disease is spreading faster to the healthy trees in the absence of the protected barrier of inoculated cacao trees. None of the cacao trees in treatment *T*_3_ was inoculated with the N1 mild strain and therefore did not have protection against the severe 1A strain. Our approach of using differential equations leads to the same results as the more elaborate approach used in Domfeh *et al.* [10].

The models used in this study focuses only on the depletion of the healthy cacao trees unlike the more complex models that describe the evolution of healthy and distinct classes of infected trees [12]. We observe that, despite the simplicity of these models, we are still able to use them to capture some of the experimental data points for treatments *T*_1_, *T*_2_, *T*_3_. We also notice that some models give better results than others, for instance, model (2) performs better than model (1), while model (8) performs better than model (9). Of course the performance of models (8) and (9) depends on the setting of the width at the onset of the simulation. There is no clear difference in the performance of the stochastic models (5) and (6) without delay. This can be attributed to the noise in the data because it is not easy to identify infected trees.

To summarize, the models with stochasticity and delay, although more complicated, are more realistic because they take into account the variability and errors in the treatment observations *T*_1_, *T*_2_, *T*_3_; and also consider the time it takes for the trees to be infected which cannot be neglected.

### 5.2 Conclusions

To conclude, in this study we have developed two different types of simple models to capture the depletion of healthy cacao trees using three treatment data *T*_1_, *T*_2_, *T*_3_ obtained from an experimental study at the Ghana Cocoa Research Institute [10]. We have found the following results summarize below

(i) Using the simple models we can estimate the transmission rate that shows the advantage of the protective layer of the mild CSSV N1 strain.
(ii) Simple deterministic models can capture the dynamics of the disease like more elaborate SIR-type model.
(iii) The models with delay perform better in capturing the dynamics of the infection obtained from the experimental data.
(iv) The models combining delay and stochasticity, although they are more complicated, they are more realistic because they take into account the variability and errors in the treatments. They also account for the time it takes for the trees to be infected which reflect more accurately the scenarios under study.

While we have used these simple models to gain informative insights about the data and the nature of disease spread, the models have drawbacks, for instance, these models cannot be used to understand the competitive nature of the strains, address vital questions related to the width of the protective layers, or describe the geometry of these layers to ensure adequate protection of the healthy trees in order to enhance the farmers yields. In our future work, we will develop appropriate models to address these questions that among legitimate concerns of every farmer.

## Supporting information

S1 Fig Stochastic model without delay and additive noise. Numerical simulations of the stochastic model (5) without delay and additive noise. Panels (a)–(c), show the results for Experimental treatment *T*_1_, *T*_2_, *T*_3_, respectively.
(TIFF)

S2 Fig Stochastic model without delay and multiplicative noise. The simulations of the model (6) for treatment *T*_1_, *T*_2_ and *T*_3_, are depicted in S2 Fig. Numerical simulations of the stochastic model (6) without delay and multiplicative noise. Panels (a)–(c), show the results for Experimental treatment *T*_1_, *T*_2_, *T*_3_, respectively.
(TIFF)
