# Supplementary figures and images for: Cacao sustainability: The case of cacao swollen-shoot virus co-infection
Source: PLoS One. 2024 Mar 7;19(3):e0294579. doi: 10.1371/journal.pone.0294579 (PMC10919592; doi:10.1371/journal.pone.0294579)

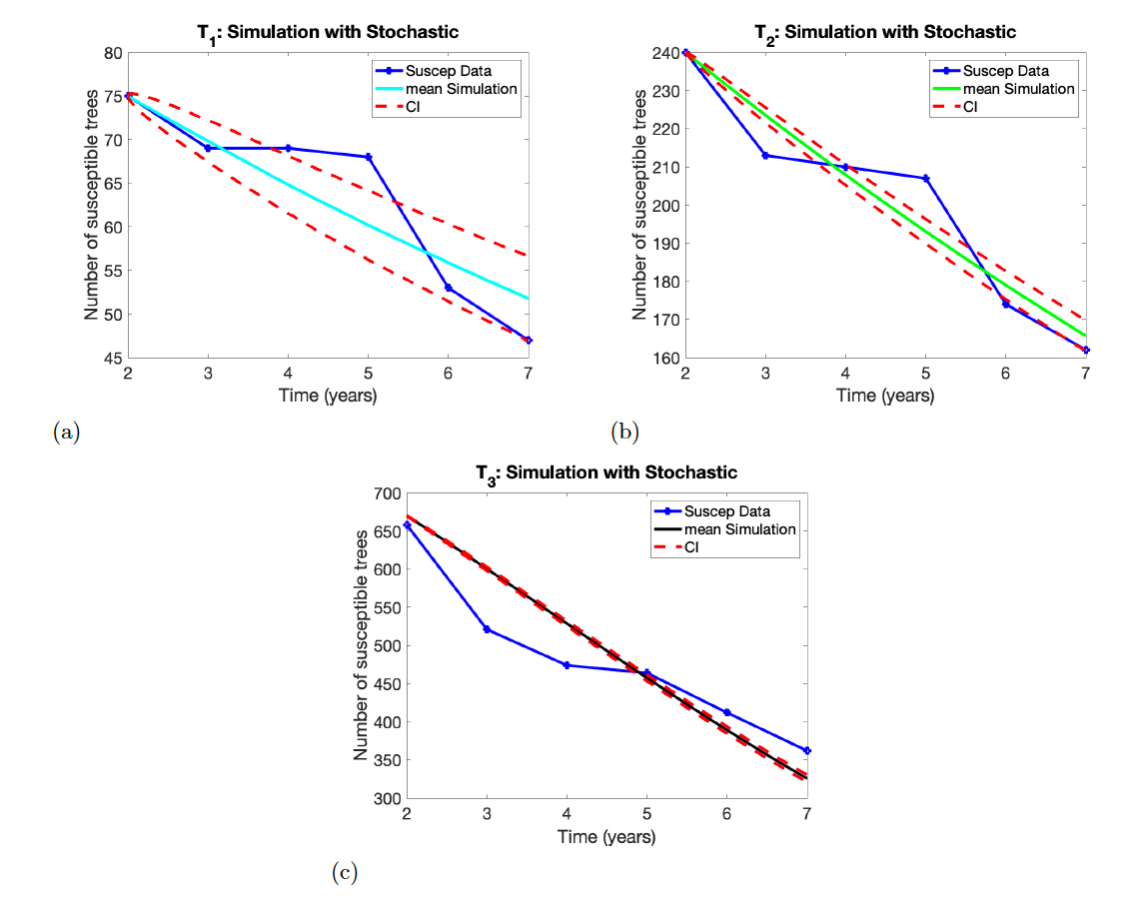

Supplement: S1 Fig — Numerical simulations of the stochastic model (5) without delay and additive noise. Panels (a)–(c), show the results for Experimental treatment T1, T2, T3, respectively. (TIFF) [file pone.0294579.s001.tiff]

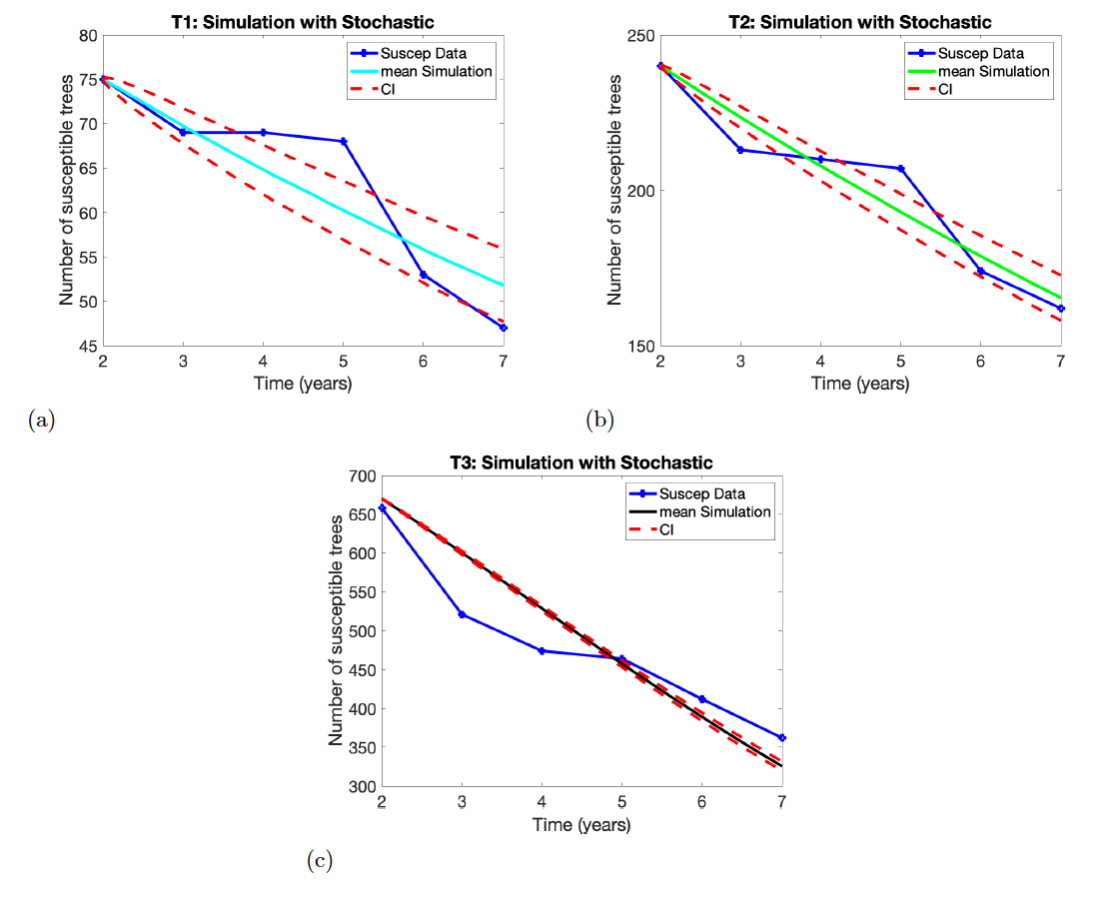

Supplement: S2 Fig — The simulations of the model (6) for treatment T1, T2 and T3, are depicted in S2 Fig. Numerical simulations of the stochastic model (6) without delay and multiplicative noise. Panels (a)–(c), show the results for Experimental treatment T1, T2, T3, respectively. (TIFF) [file pone.0294579.s002.tiff]
